# Resistance Exercise Training-Induced Muscle Hypertrophy Was Associated with Reduction of Inflammatory Markers in Elderly Women

**DOI:** 10.1155/2010/171023

**Published:** 2010-12-28

**Authors:** Kishiko Ogawa, Kiyoshi Sanada, Shuichi Machida, Mitsuharu Okutsu, Katsuhiko Suzuki

**Affiliations:** ^1^Research Team for Social Participation and Health Promotion, Tokyo Metropolitan Institute of Gerontology, 35-2 Sakaecho, Itabashi, Tokyo 173-0015, Japan; ^2^Consolidated Research Institute for Advanced Science and Medical Care, Waseda University, 513 Wasedatsurumakicho Shinjuku, Tokyo 162-0041, Japan; ^3^School of Physical Education, Tokai University, 1117 Kitakaname, Hiratsuka, Kanagawa 259-1292, Japan; ^4^Faculty of Human Sciences, Waseda University, 2-579-15 Mikajima, Tokorozawa, Saitama 359-1192, Japan

## Abstract

Aging is associated with low-grade inflammation. The benefits of regular exercise for the elderly are well established, whereas less is known about the impact of low-intensity resistance exercise on low-grade inflammation in the elderly. Twenty-one elderly women (mean age ± SD, 85.0 ± 4.5 years) participated in 12 weeks of resistance exercise training. Muscle thickness and circulating levels of C-reactive protein (CRP), serum amyloid A (SAA), heat shock protein (HSP)70, tumor necrosis factor (TNF)-*α*, interleukin (IL)-1, IL-6, monocyte chemotactic protein (MCP-1), insulin, insulin-like growth factor (IGF)-I, and vascular endothelial growth factor (VEGF) were measured before and after the exercise training. Training reduced the circulating levels of CRP, SAA (*P* < .05), HSP70, IGF-I, and insulin (*P* < .01). The training-induced reductions in CRP and TNF-*α* were significantly (*P* < .01, *P* < .05) associated with increased muscle thickness (*r* = −0.61, *r* = −0.54), respectively. None of the results were significant after applying a Bonferroni correction. Resistance training may assist in maintaining or improving muscle volume and reducing low-grade inflammation.

## 1. Introduction

 Accumulating investigations have proven that elevated levels of several circulating inflammatory mediators among apparently healthy men and women have predictive value for future adverse events [1, 2]. Prospective epidemiological studies have found increased vascular risk to be associated with increased basal levels of cytokines such as interleukin (IL)-6 and tumor necrosis factor (TNF)-*α* [3, 4] and downstream acute-phase reactants such as C-reactive protein (CRP), fibrinogen, and serum amyloid A (SAA) [5, 6]. The levels of these inflammatory markers increase as a result of local inflammation in response to an acute infection or trauma and then decrease when the infection or trauma is resolved. On the other hand, a low-level increase in the systemic concentrations of these inflammatory markers is defined as low-grade inflammation [7]. Chronic low-grade inflammation is related to atherosclerosis, which is characterized by the accumulation of lipid and fibrous elements in the large arteries [7]. Atherosclerotic plaques attract inflammatory cells, which in turn produce reactive oxygen species and inflammatory cytokines such as TNF-*α* and monocyte chemoattractant protein (MCP)-1. These inflammatory events exacerbate atherosclerosis and promote thrombosis [7]. Chronic low-grade inflammation is also related to insulin resistance. Individuals with type 2 diabetes mellitus overexpress TNF-*α* in adipose tissue; this TNF-*α* spills into the circulation, inhibiting the action of insulin in metabolically active tissue such as skeletal muscle. Increases in circulating levels of CRP, TNF-*α*, and IL-6 have been observed in the aged population [8] and may partly account for the increased risk of atherosclerosis, type 2 diabetes mellitus, hypertension, and other cardiovascular diseases [6, 9]. Inflammatory responses are initiated to restore homeostasis in response to noxious conditions such as sarcopenia, frailty and disability in aged people. While acute inflammation resolves when the trigger ceases, chronic inflammation remains in response to the homeostatic imbalance caused by chronic noxious conditions.

 Regular exercise training protects against atherosclerosis and type 2 diabetes [10, 11]; in addition, evidence exists to support a relationship between regular exercise and improvements in low-grade inflammation. In cross-sectional studies, persons who are more physically active have lower concentrations of white blood cells (WBCs), CRP, and fibrinogen [12–15]. Several longitudinal studies suggest that increasing physical activity could be effective in reducing systemic inflammation [16–18]. Experimental studies examining whether acute exercise induces the anti-inflammatory response have collectively provided support for the notion that exercise may protect against low-grade inflammation [19]. Furthermore, inflammation is associated with the age-related loss of muscle mass and muscle strength (e.g., sarcopenia) [20]. High levels of circulating inflammatory markers and/or cytokines are associated with low muscle mass in aged and obese people [20, 21]. To date, resistance exercise has not yet been shown to affect the plasma level of TNF-*α*, soluble TNF receptor (sTNFR), and IL-6 [22], but has been shown to affect skeletal muscle TNF-*α* mRNA expression [19]. In theory, IL-6 may partly contribute to the anti-inflammatory activities (e.g., decreased production of TNF-*α*) because TNF-*α* stimulates the production of IL-6. In return, IL-6 inhibits the transcription of TNF-*α* [23] and stimulates the production of anti-inflammatory cytokines and the shedding of TNF receptors that bind TNF-*α* with high affinity. An acute bout of exercise attenuated an endotoxin-induced increase in TNF-*α* in humans [24], but also decreased TNF-*α* in IL-6 knockout mice [25], suggesting that exercise may protect against chronic systemic low-grade inflammation via IL-6-independent pathways. Therefore, long-term resistance exercise in the elderly may be effective to protect against chronic systemic low-grade inflammation. 

 Whereas numerous studies confirm the benefits of regular exercise, determining the appropriate exercise intensity for aged people is an important and difficult clinical decision. It has been observed that one in five elderly subjects (older than 70 years) was unable to execute the classical treadmill-based exercise test, either due to a fear of falling or because of physical or cognitive limitations [26]. Since designated exercise protocols in intervention studies tend to be intensive, including a load equivalent to 50%–80% of maximal work rate several times per week, the impact of low-intensity exercise on low-grade inflammation in aged people may provide useful information for the design of exercise training programs and risk assessment of exercise training regimens. The aim of the present study was to examine the effect of low-intensity exercise on physical fitness, muscle thickness, and circulating markers of inflammation and growth factors in elderly women.

## 2. Methods

### 2.1. Subjects

 The present study was part of a survey for nursing home residents in Tokorozawa, Saitama, Japan conducted by Waseda University to test the effect of physical training for 12 weeks. Twenty-one elderly women (mean age, 85.0 ± 4.5 years) participated in the survey. Subjects received a medical examination, including blood pressure (Omron digital automatic blood pressure, HEM-1000; Omron Health Care, Tokyo, Japan), waist circumference (tape measure; TJM, Tokyo, Japan), weight (Inner Scan 50 V; TANITA, Tokyo, Japan), height (Digital height meter, 4D200R; Endo Electrics, Tokyo, Japan), blood tests (MBL Corporation, Tokyo, Japan), and an interview by a physician. None of the subjects had participated in any exercise training on a regular basis. The participants lived freely in the nursing home and sometimes participated in the social program provided by the nursing home. There was no physical exercise program in the nursing home during the intervention. A diet of 1600 kcal/day was provided based on Japanese nutrient intake standards. All elderly who voluntarily participated were considered eligible and were excluded only if they did not have permission to exercise by a physician. All participants were informed of the purpose and risks of the study before written informed consent was obtained. The Research Ethics Committee at Waseda University approved the study protocol.

### 2.2. Exercise Protocol

The participants took part in the exercise sessions at least once per week for 12 weeks. The exercise program involved low-intensity resistance exercise. The duration of each exercise session was approximately 40 minutes, consisting of a 5-minute warm-up period, 30 minutes of resistance exercise, and a 5-minute cool-down period. Four exercise forms, consisting of foot press, front traction, vertical traction, and shoulder press, were performed on Kinesis devices (Technogym Japan, Tokyo, Japan) designed for strength training. The Kinesis device contains elastic cables that are stretched by the subject to perform the following four exercise forms: foot press (a cable is attached to the foot from the floor, and the thigh is raised); front traction (a vertically drawn cable is grasped and stretched forward); vertical traction (a cable is grasped from above and stretched downward); shoulder press (a cable is attached to the shoulders from the floor and the shoulders are raised and lowered). The exercise intensity was progressively increased at the request of the participants. All participants were required to perform each repetition in a slow, controlled manner with a rest between sets. One set of ten repetitions was performed for the foot press, front traction, and shoulder press. Two sets of ten repetitions were performed for vertical traction. All sessions were supervised to ensure safety and to monitor the appropriate load of exercises.

### 2.3. Blood Sampling and Analysis

 Blood samples were collected before and after the 12-week intervention program (at least 17 hours after the last bout of exercise). Blood was collected in the morning at 8 to 9 am after an overnight fast. Blood was drawn from a forearm vein into a sterile tube containing heparin (Termo, Tokyo, Japan). White blood cells (WBCs), lymphocytes, and neutrophils were counted with a Sysmex microcell counter pocH-100i (TOA Medical Electronics, Kobe, Japan). Blood was centrifuged for 10 min at 1000 g to separate plasma and stored at −80°C until analysis. Routine clinical tests were performed by a hematology laboratory (MBL Corporation, Tokyo, Japan) to determine the levels of total protein (TP), total cholesterol (T-CHO), high-density lipoprotein (HDL) cholesterol, low-density lipoprotein (LDL) cholesterol, triglyceride (TG), free fatty acid (FFA), and glucose.

 Commercially available enzyme-linked immunosorbent assay (ELISA) kits were used to measure the plasma concentrations of CRP, SAA, insulin-like growth factor (IGF)-I (Diagnostic Systems Laboratories. Inc., Webster, TX, USA), vascular endothelial growth factor (VEGF) (BioSource International, Inc. Camarillo, CA, USA), heat shock protein (HSP)70 (Stressgen Biotechnologies Co., Victoria, BC, Canada), TNF-*α* (R&D Systems Inc. Minneapolis, MN, USA), IL-6 (R&D Systems Inc.), insulin (BioSource International), and monocyte chemoattractant protein (MCP)-1 (R&D Systems Inc.). The absorbance was measured spectrophotometrically with a microplate reader (VersaMax Molecular Devices, Sunnyvale, CA, USA). The plasma concentration of each marker was calculated by comparison to a standard curve established in the same measurement. Insulin resistance was calculated according to the homeostasis model assessment ratio with the following formula: [insulin concentration] × [blood glucose concentration]/405.

### 2.4. Physical Performance Test

A physical performance test that assessed muscle strength (hand grip), physical balance (one-leg stand with opened eyes), rapid motion (grasping a dropped stick), and body flexibility (anteflexion) was performed before and after the 12-week exercise training program. Muscle thickness was measured by B-mode ultrasound with a 5-MHz scanning head (SSD-500, Aloka, Tokyo, Japan) at six sites on the anatomical skeletal muscle belly including anterior and posterior upper arm, abdomen, subscapularis, and anterior and posterior thigh [27].

### 2.5. Statistical Analysis

 The data analysis was performed with Statview software 5.0.1 (SAS Institute Inc., Cary, NC, USA). *P* values less than.05 and.0024 (e.g., applying a Bonferroni correction) were considered statistically significant. Data are presented as means ± standard deviations (SD) for all values. The data were not normally distributed; therefore, the Wilcoxon signed-rank test was used to compare the values before and after the exercise training program in order to identify potential exercise-induced changes in hematological variables, physical performance, inflammatory markers, and cytokines. Associations between various parameters before and after the exercise program as well as percent changes ([postvalues – prevalues]/[prevalues] × 100) in those values were evaluated by using the Spearman correlation coefficient.

## 3. Results

The following chronic diseases were present in the subject group (as determined by the physician interview): type 2 diabetes (*n* = 2), hypertension (*n* = 5), heart disease (*n* = 1), chronic kidney disease (*n* = 1), and other comorbidities (*n* = 2). The other clinical characteristics of the study subjects are shown in Table 1. The participants took part in the exercise sessions at least once per week for 12 weeks. The average total number of sessions over the training period was 14.4 ± 3.7 (mean ± SD); some participants were highly motivated and completed more than 12 sessions.

 Systolic blood pressure (SBP) (*P* < .05), WBC (*P* < .05), T-CHO (*P* < .01), TG (*P* < .05), insulin (*P* < .05), and insulin resistance (*P* < .05) were significantly decreased after the training, but body weight, BMI, waist circumference, DBP, HDL cholesterol, LDL cholesterol, FFA, and glucose did not change significantly after the training (Table 1). There were no significant changes after applying a Bonferroni correction.

 The 12-week physical training did not significantly improve physical performance. However, balance, rapid movement, and body flexibility showed some improvement after the training (Table 2). The muscle thickness of the posterior upper arm (*P* < .01), abdomen (*P* < .01) and subscapularis (*P* < .05) was increased after the training, whereas the thickness of the anterior upper arm, anterior thigh and posterior thigh did not change (Table 2). There were no significant increases after applying a Bonferroni correction.

 The plasma concentrations of CRP (*P* < .05), SAA (*P* < .05), HSP70 (*P* < .01), and IGF-I (*P* < .01) were significantly decreased after the 12-week training program when compared to the baseline levels but, there were no significant changes in IL-6, TNF-*α*, MCP-1, and VEGF after the training program (Table 3). None of the changes were significant after applying a Bonferroni correction.

**Figure 1:**
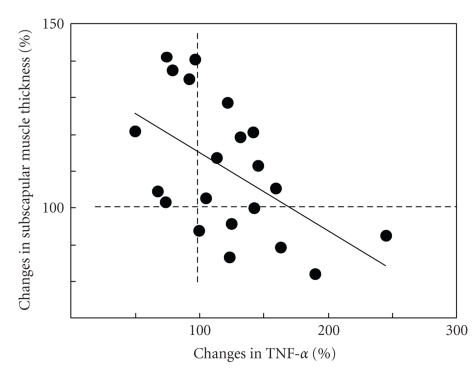
Correlation between percent change in TNF-*α* and subscapular muscle thickness *r* = − 0.54, *P* = .011. Dotted lines (100%) represent the baseline value for each subject. A greater absolute increase would appear above 100%, and a greater absolute decrease would appear below 100%. Percentage change is calculated as [postvalues – prevalues]/[prevalues] × 100.

**Figure 2:**
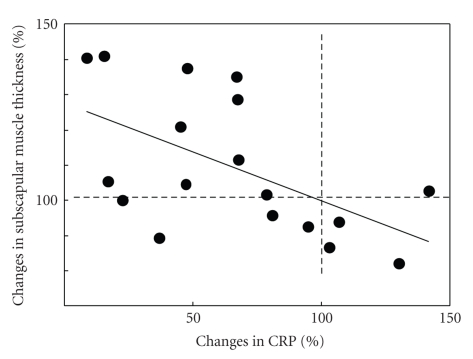
Correlation between percent change in CRP and subscapular muscle thickness *r* = −0.61, *P* = .008. Dotted lines (100%) represent the baseline value for each subject. A greater absolute increase would appear above 100%, and a greater absolute decrease would appear below 100%. Percentage change is calculated as [postvalues – prevalues]/[prevalues] × 100.

 Finally we examined the association between the change in muscle thickness following exercise training with changes in inflammatory markers and cytokines. The percent change in TNF-*α* was negatively correlated with percent change in subscapular muscle thickness (*r* = −0.54, *P* < .05) (Figure 1). The percent change in CRP was also negatively correlated with subscapular muscle thickness (*r* = −0.61, *P* < .01) (Figure 2). No significant correlations were detected in after applying a Bonferroni correction. The findings indicate that an increase in muscle thickness is associated with the decrease in TNF-*α* and CRP.

## 4. Discussion

We found that the 12-week, low-intensity, resistance exercise training program improved metabolic factors, such as blood pressure, lipids (e.g., total cholesterol and triglyceride) and insulin, muscle hypertrophy, and inflammatory markers in aged sedentary women. These results suggest that even low-intensity exercise is beneficial for aged sedentary women. Furthermore, the inverse correlation between percentage changes in subscapular muscle thickness and percentage changes in CRP and TNF-*α* suggests that exercise training-induced muscle hypertrophy is associated with reduced levels of inflammatory markers and cytokines; this finding supports the hypothesis that low-intensity exercise training is effective in reducing chronic inflammation in elderly, sedentary women.

 Inflammation may be associated with the age-related loss of muscle mass and strength [28]. Loss of skeletal muscle is the major factor that contributes to frailty and has a profound impact on the quality of life of older people [28]. TNF-*α* inhibits myogenic differentiation [29] and promotes protein catabolism [29]. In addition, TNF-*α* reduces skeletal muscle protein synthesis by decreasing translation initiation [30]. In the present study, the training program induced increased muscle thickness in the upper arm, abdomen, and subscapularis. In particular, the absolute percent change in subscapular muscle thickness (as compared to the other muscles) was associated with a reduction in inflammatory markers. Ultimately, these findings may reflect the focus on arm-specific exercises within this intervention program.

 The circulating levels of CRP, SAA, and HSP70 decreased in elderly women in response to the present exercise training program (Table 3). SAA is produced by hepatocytes and secreted into the serum as well as CRP [31]. Both SAA and CRP show a strong independent relationship with future cardiovascular events [6, 32, 33]. Although the physiological significance of SAA remains controversial, some studies have demonstrated that SAA associates with HDL-cholesterol to remove it from the site of inflammation [31]. In the present study, the levels of HDL-cholesterol did not change after the exercise training program, and we did not find a correlation between HDL-cholesterol and SAA or CRP. This discrepancy may be due to the smaller sample size of the present study. Therefore, larger more diverse studies assessing changes in HDL-cholesterol and its relationship with CRP and SAA following exercise training are warranted.

In previous studies, serum HSP70 concentrations were found to be elevated in patients with hypertension [34], peripheral and renal vascular disease [35], cerebral ischemia [36], intermittent claudication [37], and critical lower limb ischemia [37]. The serum HSP70 concentration decreased following a 14-week cardiac rehabilitation therapy, statin treatment, and a combination therapy of rehabilitation and statin for patients with coronary artery disease [38]. Therefore, we interpret the reduction of HSP70 in our study as an indication that exercise training reduces inflammation. The mechanism by which circulating levels of HSP70 (e.g., extracellular HSP70) are involved in systemic low-grade inflammation is not known. While HSPs are leaked into the extracellular compartment due to necrotic cell death [39], HSP70 can be released independently of necrotic cell death in response to a number of stressful conditions including exhaustive exercise [40]. Future investigations will explore the physiological significance of extracellular HSPs.

Growth factors such as insulin-like growth factors (IGFs) are related to insulin and mediate many actions of growth hormone, including promotion of myoblast proliferation, differentiation, and protein accumulation in muscle. Serum IGF-I levels decrease with age and are regarded as a potential mediator of sarcopenia [41]. Therefore, IGF-I levels may reflect distal outcomes of clinical importance in the elderly, such as objective performance on muscle-dependent tasks and mobility in the elderly. However, in the present study, the level of plasma IGF-I decreased after the exercise program. The results from prospective training studies are controversial, as the levels of IGF-I in different studies have been elevated [42] or reduced [43, 44] following a strenuous training program for 4–11 weeks in young individuals. Future investigations will explore the physiological significance and interpretation of the exercise training—induced reduction on circulating levels of IGF-I.

The limitations of the present study were the lack of control group, small sample size, and no records for the daily physical activity outside of the sessions. Considering that the average number of sessions (14.4 ± 3.7) was more than 12, the results may be due to the motivation of the participants to exercise. The participants were encouraged to increase their physical activity during the intervention period. The number of daily steps was recorded for some of the participants (*n* = 10). There was an increase in the average number of daily footsteps from the 1st week (2100 steps per day) to the 12th week (3000 steps per day). Also, the participants enjoyed their interactions with the young instructors and college students who visited and talked with them every week during the exercise sessions. Thus, there might be a physiological confounding factor in the increase in daily activities that contributed to the reduction of these metabolic factors. In addition, none of the results were significant after applying a Bonferroni correction. It is possible that there were spurious positive tests due to the large number of statistical comparisons performed.

## 5. Conclusion

In conclusion, our data further support the beneficial role of exercise training in elderly women as a valid strategy to improve blood pressure, insulin resistance, and muscle mass, and reduce the circulating levels of CRP, SAA, and HSP70. Our data provide additional information on the design of an exercise training program for preventing sarcopenia in aged people and the risk assessment of exercise training regimens. Understanding the association between exercise, nutrition, and inflammatory biomarkers is essential given the public health importance of frailty and sarcopenia with ageing.

##  Conflict of Interests

All authors declare that there is no conflict of interests.

##  Authors Contributions

K. Ogawa, K. Suzuki conceived and designed the investigation. K. Ogawa, K. Suzuki, S. Machida, and M. Okutsu performed the experiments. K. Ogawa analyzed the data, and K. Suzuki contributed the reagents/materials/analysis tools. K. Ogawa wrote the paper.

## Figures and Tables

**Table 1:** Change in anthropometric characteristics and biochemical factors before and after exercise training in elderly women (*n* = 21).

	Pretraining	Posttraining
	Mean ± SD	Mean ± SD
Weight (kg)	46.3 ± 9.8	46.9 ± 9.3
BMI (Body Mass Index)	21.2 ± 4.0	21.3 ± 3.8
Waist circumference (cm)	80.4 ± 11.3	79.9 ± 11.2
Systolic blood pressure (mm Hg)	155.4 ± 19.7	145.5 ± 15.7*
Diastolic blood pressure (mm Hg)	76.6 ± 11.3	72.2 ± 12.4
White blood cells (10^5^ cells·mL^−1^)	59.2 ± 4.4	48.8 ± 2.2*
Total cholesterol (g·L^−1^)	217.2 ± 10.5	202.7 ± 9.7**
HDL-cholesterol (g·L^−1^)	62.2 ± 3.7	59.8 ± 3.4
LDL-cholesterol (g·L^−1^)	119.4 ± 7.8	115.0 ± 8.0
Free fatty acid (mEQ·L^−1^)	0.5 ± 0.1	0.6 ± 0.1
Triglyceride (g·L^−1^)	102.9 ± 13.4	91.9 ± 12.1*
Glucose(mg·dL^−1^)	104.4 ± 8.2	96.4 ± 5.1
Insulin (*μ*U·mL^−1^)	8.3 ± 2.2	4.3 ± 1.1*
Insulin resistance (HOMA-R)	2.3 ± 0.7	1.1 ± 0.3

*P < .05 as determined by the Wilcoxon signed-rank test.

**P < .01 as determined by the Wilcoxon signed-rank test.

HDL; High density lipoprotein, LDL; Low density lipoprotein.

Insulin resistance = [insulin concentration] × [blood glucose concentration]/405.

**Table 2:** Changes in physical performance and muscle thickness pre- and post exercise training in elderly women (*n* = 21).

	Pre	Post
	mean ± SD	mean ± SD
*Physical performances*		
Muscle strength (kg) (hand grip)	17.2 ± 3.6	16.3 ± 3.7
Balance (sec) (one leg stand with closing eyes)	3.0 ± 1.8	3.5 ± 3.0
Rapid movement (cm) (grasping the dropped stick)	27.4 ± 9.8	25.5 ± 7.4
Flexibility (cm) (anteflexion)	27.7 ± 9.4	28.0 ± 7.8

*Muscle thickness (mm)*		
Anterior upper arm	208 ± 24	205 ± 24
Posterior upper arm	187 ± 34	221 ± 43**
Abdomen	66 ± 18	70 ± 20**
Subscapula	142 ± 27	159 ± 34*
Anterior thigh	326 ± 54	317 ± 52
Posterior thigh	492 ± 62	480 ± 65

*Significant differences between Pre and Post values. *P* < .05 Wilcoxon was used as the statistical analysis.

**Significant differences between Pre and Post values. *P* < .01 Wilcoxon was used as the statistical analysis.

**Table 3:** Changes in inflammatory markers pre and post exercise training in elderly women (*n* = 21).

	Pre	Post
	mean ± SD	mean ± SD
CRP (ng·mL^−1^)	2.44 ± 3.22	1.46 ± 2.22*
SAA (*μ*g·mL^−1^)	54.2 ± 39.7	34.9 ± 35.1*
HSP70 (ng·mL^−1^)	3.3 ± 2.1	2.2 ± 1.7**
IL-6 (pg·mL^−1^)	1.80 ± 2.18	1.95 ± 2.57
TNF-*α* (pg·mL^−1^)	0.91 ± 0.65	1.04 ± 0.79
MCP-1 (pg·mL^−1^)	196 ± 76	198 ± 61
IGF-1 (ng·mL^−1^)	213 ± 80	144 ± 46**
VEGF (pg·mL^−1^)	33 ± 7	34 ± 8

*Significant differences between Pre and Post values. *P* < .05 Wilcoxon was used as the statistical analysis.

**Significant differences between Pre and Post values. *P* < .01 Wilcoxon was used as the statistical analysis.

CRP: C-reactive protein, SAA: serum amyloid A, HSP: heat shock protein, IL-6: interleukin-6, TNF: tumor necrosis factor, MCP: monocyte chemotactic protein, IGF: insulin like growth factor, and VEGF: vascular endothelial growth factor.
